# Are We Meeting the Demand for Pharmacist-Led Antimicrobial Stewardship Programs during Postgraduate Training-Year 1 (PGY1)?

**DOI:** 10.3390/pharmacy8020091

**Published:** 2020-05-27

**Authors:** Edoabasi U. McGee, Arrington D. Mason-Callaway, Brent L. Rollins

**Affiliations:** School of Pharmacy, Philadelphia College of Osteopathic Medicine–Georgia Campus, Suwanee, GA 30024, USA; arringtonma@pcom.edu (A.D.M.-C.); brentro@pcom.edu (B.L.R.)

**Keywords:** antimicrobial, stewardship, postgraduate, residency, training, pharmacist, infectious, disease

## Abstract

In the United States of America, pharmacists play a pivotal role in antimicrobial stewardship; training from postgraduate residency may hone knowledge and skills gained from didactic pharmacy education. Specifically, the first year of postgraduate training, the learner may become an “everyday steward in training” and may go on to complete a second year in infectious diseases. However, there are a limited number of second year infectious diseases programs. The current demand for pharmacist to participate in and or lead stewardship is disproportionate to available specialized training. The first year of post-graduate training has to be setup to ensure appropriate preparation, so newly trained pharmacist may help meet the demand. Currently, no clear standards exist for training in the first year. The purpose of this study is to survey the nature of stewardship training performed by first year residents from the perspective of residency program directors and preceptors. A 13-question online survey was distributed to examine resident exposure to antimicrobial stewardship activities. Survey data from targeted residency directors and preceptors were analyzed. A third of the programs required it as a mandatory rotation. Resident’s stewardship activities ranged from program to program; there was not consensus of the training activities.

## 1. Introduction

A major public health crisis affecting the world today is the growing number of resistant strains of bacteria, which can develop from inappropriate use of antimicrobials [1,2]. In the United States of America, approximately 30–50% of antimicrobial use in hospitals and approximately 30% of antimicrobials prescribed in the ambulatory care setting are either unnecessary or inappropriate [3,4]. Consequentially, more than 2.8 million antibiotic-resistant infections occur in the United States of America each year, and more than 35,000 people die as a result [5]. O’Neil and colleagues completed a review on antimicrobial resistance, and projected that by 2050, approximately more than 10 million people will die annually from resistant infections [6]. One major solution that has been recommended to address this crisis is antimicrobial stewardship. It is the keystone to improving patient safety, minimizing antibiotic resistance and Clostridioides difficile [3]. Antimicrobial stewardship programs (ASPs) with pharmacist leadership have been shown to improve patient outcomes, reduce rates of resistance, and decrease healthcare costs [7]. In the United States of America, pharmacy leadership in ASPs is supported by one of the seven core elements established by the Center for Disease Control and Prevention (CDC) [8]. The core elements include leadership commitment, accountability, drug expertise, action to support optimal use, tracking/monitoring, reporting, and education [8]. The element of drug expertise involves a single appointed pharmacist leader with proficient knowledge in infectious diseases leading ASPs in collaboration with an infectious diseases physician. Training of pharmacist with the skills required to lead or participate in ASP has not been distinctly defined.

Pharmacy residency training is critical for pharmacists that will participate in direct patient care activities, such as is seen in ASP. In the United States of America, residency training occurs after completion of a pharmacy degree, though only a degree is required for licensing of a pharmacist. The American College of Clinical Pharmacist (ACCP)-Council on Education and Workforce Development established a goal [9]. By 2020, all new college of pharmacy graduates who will be providing direct patient care will be required to complete an American Society of Health System Pharmacist (ASHP)-accredited postgraduate-year-one (PGY1) residency [9]. To accomplish this goal, ASHP increased the number of ASHP-accredited training programs and positions since the establishment of the goal. To date, over 1400 PGY1 programs are ASHP-accredited residencies [10]. Post-graduate residency training may involve one or two years. Training involves increasing knowledge and experiences for pharmacist to further hone direct patient care skills. The goal of the first year is to develop general competencies in managing medication use systems and supporting optimal medication therapy outcomes for patients with a wide range of diseases. The goal of the second year or postgraduate year two (PGY2) is to focus on a specialized area of practice. Currently over 1200 PGY2 programs are ASHP accredited with varied specializations [10].

The Joint Commission, a healthcare organization accrediting body recently released a mandate (effective 1 January 2020) requiring ambulatory care centers to establish ASPs to maintain accreditation [11]. This mandate is closely in line with the antimicrobial stewardship standard that went into effect 1 January 2017 for hospitals and critical access hospitals [12]. This mandate ultimately increased the need for ASP pharmacists to be trained and available to lead or participate in stewardship. The Infectious Diseases Society of America (IDSA) and the Society of Healthcare Epidemiology of America (SHEA) define an ASP pharmacist as one that has completed an accredited PGY1 residency, and an accredited PGY2 residency in infectious diseases [13,14]. Recommendations for the training and certification of pharmacists in infectious diseases pharmacotherapy provided by the Society of Infectious Diseases Pharmacists (SIDP) and the IDSA state that PGY1 programs only offer a rudimentary introduction to infectious diseases, owing to the short term nature of the training [14]. As of 2019, a total of 3348 positions were matched for PGY1 programs, and only 114 infectious diseases PGY2 ASHP-accredited programs were available [15]. Due to the small number of infectious diseases PGY2 training programs, a potential “bridge” to fill that gap must be explored to ensure that the alarming projection made by O’Neil and colleagues regarding significant increases in mortality from antibiotic resistant infections in the future is delayed.

Various studies based in the United States of America have shown the use of advanced pharmacy practice experience (APPE) student pharmacists, PGY1 and PGY2 pharmacy trainees in conducting antimicrobial stewardship. Benson and colleagues utilized APPE students in a long-term acute-care hospital to perform prospective audit with intervention and feedback, dose optimization, and streamlining/de-escalation of therapy [16], while Siegfried and colleagues utilized PGY2 residents (infectious diseases and critical care) in an academic medical center to perform prospective audit with intervention and feedback, formulary restriction and preauthorization, and dose optimization [17]. Kufel and colleagues evaluated the extent, content, and methodology by which ASP education was incorporated into the curricula of colleges and schools of pharmacy in the United States of America [18]. The study found variable results in the curriculum, and recommended increased exposure due to the demand on pharmacists needed to perform antimicrobial stewardship.

In addition to curricular changes within colleges and schools of pharmacy in the United States, it is imperative that advanced training in antimicrobial stewardship principles during PGY1 training be performed to equip pharmacists to lead and or participate in the charge of ASPs, and meet the demands set forth by the Joint Commission. Additionally, the ASHP accreditation standard for PGY1 pharmacy residency programs standard 3.2.b. (1)–(4) are considered critical goals [19]. Specifically, the program’s educational goals and objectives must support achievement of advancing practice and improving patient care [19]. By receiving adequate education and learning experiences, trainees advance the practice and significantly impact patient outcomes within ASPs. With the limited number of infectious diseases PGY2 training programs, and this increased demand set forth by the Joint Commission, appropriate training must occur in the PGY1 year to equip pharmacists with the advanced knowledge and experience necessary to effectively participate and or lead ASPs. Since there are significantly more PGY1 programs than infectious diseases PGY2 programs, training these individuals during the PGY1 year may be very beneficial. The literature to date has focused on implementation of various ASPs utilizing trainees at individual centers. However, the standardization of ASP training for PGY1 pharmacy trainees has not been established. To date, no study has surveyed the overall nature of ASP training performed by PGY1 programs. The purpose of this study is to characterize the training activities of PGY1 pharmacy residents in antimicrobial stewardship from the perspective of residency program directors and preceptors.

## 2. Materials and Methods 

This was a descriptive cross-sectional study utilizing an electronic survey questionnaire designed to further characterize the antimicrobial stewardship activities in which residents were exposed to during their first postgraduate training year. The study was approved by the institutional review board of the Philadelphia College of Osteopathic Medicine. The questionnaire was sent to PGY1 residency program directors (RPDs) and/or infectious diseases preceptors via Qualitrics^®^ online software. All RPDs for health-system pharmacy PGY1 programs listed in the ASHP residency directory as of January 2016 were included. In addition, infectious diseases preceptors were also contacted using the Practice Research Network (ID-PRN) group listserv for the American College of Clinical Pharmacists (ACCP) in January 2016. The ID-PRN participants were given instructions to only complete the survey once if they were both a PGY1 residency director and a member of the ACCP ID PRN listserv. PGY1 programs that were community-based and managed care-based pharmacy residencies were excluded, and programs with lack of reliable email contacts were excluded.

The survey consisted of 13 questions. For questions pertaining to PGY1 ASP activities, participants were asked to use a 4-point Likert-scale (1 = never, 2 = sometimes, 3 = often [weekly], 4 = always [daily]). Questions were related to demographics of the residency program (total number of questions = 7), participants’ perception of Joint Commission ASP mandate (total number of questions = 3), PGY1 ASP activities (total number of questions = 3). Table 1 further depicts all 13-survey questions. Participants were given one month to complete the survey, and one reminder email was sent two weeks prior to survey closing. The survey instrument was developed using the framework of the CDC guidelines on “The Core Elements of Hospital Antibiotic Stewardship” by infectious diseases pharmacists with practice experience in antimicrobial stewardship [8]. The survey was piloted among pharmacy practice faculty at the Philadelphia College of Osteopathic Medicine, to include one PGY1 RPD. Edits were made to revise the language and delivery of questions based on feedback. Surveys that were fully completed were included in the analysis. There were a total of 2799 RPDs and ACCP ID PRN listserv initially evaluated to participate in the study, but 2332 were excluded due to lack of reliable email contact information. Therefore, the total number of participants included in the study was 467. The results of the primary outcome were evaluated using descriptive statistics (mean and standard deviation [S.D.]).

## 3. Results

Of the 467 targeted residency directors and preceptors contacted, 76 participated in the survey and were therefore included in the analysis. Table 2 further describes the demographics of the survey respondents. Table 3 depicts the PGY1 program characteristics.

Approximately 33% of programs had antimicrobial stewardship as a requirement, 24% had antimicrobial stewardship as an elective, and 43% incorporated antimicrobial stewardship as part of the infectious diseases rotation. The most common daily activity performed by residents was renal dose adjustment. Table 4 further depicts the five most common daily activities performed by residents based on the type of institution.

Overall, the most tracked intervention as documented by respondents was giving prescribers direct communication concerning antimicrobial prescribing. Table 5 further describes the top five daily tracked stewardship interventions.

## 4. Discussion

Among the survey respondents, approximately one third of PGY1 programs required antimicrobial stewardship as a mandatory rotation to complete training. Most programs offered a four-week rotation. This may confirm the prior statement made by the SIDP/IDSA review that “PGY1s are broadly focused on short term training experiences, and are able to provide only a rudimentary introduction to infectious diseases [14].” Therefore, a longitudinal model for PGY1 programs may be more favorable to ensure a greater depth of experience. Community hospital programs had the highest response rate, which may indicate interest in facilities with smaller pharmacy staff to optimize their potential through the utilization of PGY1 residents to run ASPs. The programs with a bed size of 200–399 allowed their residents to perform the most types of ASP interventions, which may suggest that medium-sized facilities are utilizing their PGY1 residents more actively in performing stewardship interventions as compared to larger academic institutions that may have an ID-trained clinical pharmacist performing most duties. The top five daily activities performed by residents consist of approaches that the IDSA guidelines recommend. However, not all programs universally allow their residents to perform these approaches, including dose optimization, streamlining/de-escalation, and intravenous to oral conversion, strategies that are considered the “bread and butter” of ASPs. This potentially may highlight the lack of consensus in what the structure of training should be. While no two hospital ASPs are alike, standardization is necessary to achieve the fundamental level of competencies. The accrediting organization should consider reviewing and revising requirements for ASP training during the PGY1 year. The basic training of PGY1 residents would not serve as an outright substitute for a PGY2 infectious disease residency, but it may provide pharmacists with the core competencies and knowledge base sufficient to participate and or implement a modest ASP. In addition, ASP training certificates such as the SIDP and the Making a Difference in Infectious Diseases (MAD-ID) certifications may be given to the newly-trained pharmacist to facilitate implementing an ASP post-residency.

The full impact of the new mandatory requirement for ASP has yet to be fully ascertained in the United States of America. Gong and colleagues compared using three active ASP strategies in China to include prior authorization, audit and feedback, and pay for performance to determine the incremental effect of financially punished audit and feedback [20]. In this study, the ASP team conducted daily prior authorization and daily audit and feedback, then monthly feedback to administrative groups with imposed financial penalties and public reporting. Their findings suggest that financially punished audit and feedback is capable of catalyzing improvement in efforts on appropriate use of antibiotics in a pediatric hospital that already engaged in only prior authorization. Therefore, one can hypothesize that as accrediting bodies mandate the regulation put in place 1 January 2020 requiring ambulatory care centers to establish ASPs to maintain accreditation and the 1 January 2017 decree for hospitals and critical access hospitals to establish and maintain ASP; the shift in manpower requirements will become very transparent [11,12]. Echevarria and colleagues describe the development and validation of a staffing calculator and its use in creating staffing guidance for ASPs in Veterans Health Adminstration (VHA) facilities in the United States of America [21]. They put together a work group of the VHA pharmacy benefit management and clinical pharmacy practice that developed a list of expected ASP activities for hospitalized and long-term care patients [21]. The patient care activities were derived from published ASP guidelines and literature [21]. Ultimately, creating an objective staffing calculator that estimated staffing needs based on the volume of antibiotic orders, and program management activities that can be used to prioritize which activities to implement [21]. They found that, in order to implement and manage a robust ASP, a pharmacist full time equivalent (FTE) investment of 1.0 per 100 occupied beds would be needed [21]. Therefore, most institutions in the United States of America with at least 100 or more occupied beds would benefit from having 1.0 FTE based on this validated staffing calculator. Thus, the expected impact of the mandatory ASP regulation will increase the need for inpatient and outpatient pharmacists to lead and or participate in ASP.

To date, this is the first descriptive survey study characterizing the exposure of antimicrobial stewardship training for PGY1 pharmacy residents from the RPDs’ perspective. With the current mandate, facilities without an infectious diseases pharmacist may be unable to accomplish the goals of antimicrobial stewardship. However, clinical pharmacists may be able to participate and or lead stewardship efforts—newly-trained PGY1 pharmacists, especially, may fill this gap. Gallagher and colleagues published a commentary discussing recommendations for and examples of best practices in training student pharmacists to become antimicrobial stewards [22]. The group developed an antimicrobial stewardship knowledge pyramid [22]. It described a hierarchy of knowledge and skills for pharmacists at three levels of training/practice [22]. Knowledge and skills related to stewardship are acquired over the entire arc of a pharmacist’s career, progressing based on the training and specialization a pharmacist pursues [22]. PGY1 residents are described, as “everyday-stewards-in-training” [22]. Nevertheless, consensus on activities PGY1 residents should perform during their training to ensure they become “everyday-stewards-in training” does not currently exists. Laible and colleagues described utilizing PGY1 residents along with PGY2 residents and pharmacy students to conduct prospective audit with intervention and feedback, dose optimization, and streamlining/de-escalation of therapy [23]. Smith and colleagues described utilizing PGY1 residents along with PGY2 residents to conduct dose optimization and ASP education [24]. The literature shows a wide range of stewardship strategies that PGY1 trainees are involved in during their residency. However, further studies are needed to define the fundamental components of ASP training for PGY1 residents to help devise a standardized approach that may potentially be used by residency accrediting bodies to formalize the experiential development of future ASP leaders. 

Laguio-Vila and colleagues issued a call to action for more antimicrobial stewardship training opportunities during internal medicine and family practice residency [25]. The team claims that since non-ID trained providers prescribe most antibiotics, therefore all future providers should have an opportunity for formal ASP training [25]. The main aims of their study were to enhance antibiotic education, to allow residents to be stewards and help them gain the ability to critically assess antibiotic prescribed. The residents also learned the approach of performing an ASP quality improvement initiative. The rotation allowed residents to emerge themselves into ASP, performing prospective audit and feedback to drug use evaluations and microbiology rounds. The authors recommended making the rotation for internal medicine and family practice residents mandatory to best engage and teach them core stewardship principles. Though this study pertains to medical residents, a similar recommendation may be made for PGY1 residents; ensuring pharmacy trainees are prepared to bridge the gap and meet the demand to lead and or participate in ASP.There are a few limitations to this study. The study focused on descriptive stats and it was conducted in 2016 when ASP’s were not officially a CMS mandate, therefore programs currently may have more robust ASPs in place at their institutions that allow for more trainee involvement. However, consensus on the optimal scope and scale of “involvement” is still vague. Moreover, the observed low response rate might be a limitation to accurately assessing the current landscape of PGY1 training. Though the responders were distributed around various states all over the United States of America, the response does not capture all the PGY1 programs in the country. With that said, based on the lack of current literature concerning the training of residents in antimicrobial stewardship, it is still imperative to critically assess the findings of this report. Further studies that also incorporate a thorough analysis and understanding of individual ASP metrics, such as days of therapy (DOT) and standardized antimicrobial administration ratio (SAAR), may help inform how effective a program may be, if one can tease out metrics achieved by utilization of residents. Furthermore, performing a survey to assess resident competency and satisfaction with ASP training would also be significant to ensure the effectiveness of training.

## 5. Conclusions

There is no clear consensus on the standardized training requirements for PGY1 pharmacy residents in antimicrobial stewardship. The residency accrediting bodies may need to put together a standardized set of training requirements that mirrors typical ASP activities in current published guidelines and literature. This may serve as a call to action for SIDP to collaborate with ASHP to develop a standard for the PGY1 program to ensure infectious diseases pharmacist experts are still leading the charge on developing “everyday stewards-in-training”. Future studies are needed to further facilitate the standardization of ASP training in PGY1 programs. In the United States of America, PGY1 training should incorporate ASP training due to the standards instituted by the Joint Commission in order to meet the increased workforce demand, ensure that competent pharmacists are effectively participating in and or leading the effort to expand ASPs, and ultimately improve patient outcomes and curtail the emerging public health threat of antimicrobial resistance.

## Figures and Tables

**Table 1:** Survey Question.

Demographic Questions: 1. How many PGY1 residents do you currently have? 2. Is there currently, a formalized (i.e., a single pharmacist who is designated to lead the program) antimicrobial stewardship program (ASP) at your institution? 3. What type of setting is the Institution that houses your PGY1 program? 4. If in a hospital setting, how many beds? 5. How long has your PGY1 program been established? 6. How many pharmacist(s) are involved with your ASP program? 7. What is the training of the ID/ASP rotation preceptor?
Participants Perception of Joint Commission Mandate by the United States of America. 8. Please answer the following concerning your knowledge about the requirements for ASP. You are aware of the: regulatory requirements related to ASP. You are aware of the legislation related to ASP. 9. Does your facility have the following: A formal, written statement of support from leadership that supports efforts to improve antibiotic use (antibiotic stewardship). Receive budgeted financial support for antibiotic stewardship activities (e.g., support for salary, training, or IT support). A physician leader responsible for program outcomes of stewardship activities at your facility. 10. Is your institution is well prepared for the accreditation organizations related to antimicrobial stewardship.
PGY1 ASP activities 11. What is the requirement for your resident, with selecting ASP as a rotation? 12. Please indicate how often your resident participates in ASP task/activities. Interventions on prescribing (i.e., order entry clarification on antibiotic indications) Interventions on appropriateness of all antibiotics 48 h after the initial orders. (e.g., antibiotic time out). Approving specified antibiotics along with the ASP physician or pharmacist prior to dispensing (i.e., pre-authorization). Reviewing courses of therapy for specified antibiotic agents (i.e., prospective audit with feedback) at your facility. Changing from intravenous to oral antibiotic therapy in appropriate situations. Dose adjustments in cases of organ dysfunction (e.g., renal dosing). Dose optimization (pharmacokinetics/pharmacodynamics) to optimize the treatment of organisms with reduced susceptibility. Interventions on duplicative unnecessary orders (e.g., double anaerobic therapy with metronidazole and meropenem with negative c.diff). Interventions on time-sensitive automatic stop orders for specified antibiotic prescriptions. Interventions on policies in place to ensure optimal use of antibiotics to treat common infections (e.g., Community-acquired pneumonia, Urinary tract infection, Skin and soft tissue infections). 13. Please indicate how often your resident collect or track data for ASP interventions. Monitoring adherence to a documentation policy (dose, duration, and indication). Monitoring adherence to facility-specific treatment recommendations. Monitoring compliance of the specific interventions in place. Tracking rates of °C. difficile infection. Producing an antibiogram (cumulative antibiotic susceptibility report). Monitoring antibiotic use using one of the following metrics: days of therapy, defined daily dose, purchasing costs. Reporting facility-specific reports on antibiotic use with prescribers. Giving prescribers direct, personalized communication about how they can improve their antibiotic prescribing.

**Table 2:** Demographics of Survey Respondents, (*n* = 76).

**Hospital Setting**	
Community Hospital	44 (57.9%)
Academic	27 (36.6%)
Veterans Affair	5 (6.7%)
**Bed Size**	
>600	13 (17.1%)
400–599	25 (32.9%)
201–399	28 (36.8%)
100–199	7 (9.2%)
<99	3 (4%)

**Table 3:** PGY1 program characteristics, (*n* = 76).

**Number of Residents**	
1–4	62 (81.6%)
5–10	11 (14.5%)
>10	3 (4%)
**Years of Established Residency**	
1–5 years	18 (24%)
6–10 years	21 (28%)
11–15 years	19 (25%)
>15 years	18 (24%)
**PGY1 ASP Rotation Length**	
4-week	43 (56.6%)
5-week	14 (18.4%)
6-week	8 (10.5%)
Longitudinal	11 (14.5%)
**Number of Preceptors Involved with ASP**	
1 preceptor	37 (49%)
2–3 preceptors	29 (38%)
>4 preceptors	10 (13%)

**Table 4:** Top Five Daily Activities Performed by Residents Based on Type of Hospital.

Activities Performed by Residents	Total (*n* = 76)	Community Hospital (*n* = 44)	Academic Teaching Hospital (*n* = 27)	Veterans Affair (*n* = 5)
Renal dosing	58 (76%)	35 (80%)	19 (70%)	4 (80%)
Dose optimization	46 (61%)	25 (57%)	18 (67%)	3 (60%)
Prospective antibiotic review	44 (58%)	29 (66%)	12 (44%)	3 (60%)
Intravenous (IV) to oral (PO) conversion	40 (53%)	27 (61%)	10 (37%)	3 (60%)
Interventions on duplicate or unnecessary orders	38 (50%)	24 (55%)	11 (41%)	3 (60%)

**Table 5:** The Top Five Daily Tracked ASP Interventions.

Giving prescribers direct communication concerning their antimicrobial prescribing	18 (24%)
Compliance monitoring of specific interventions in place	16 (21%)
Adherence monitoring to facility specific treatment recommendations	14 (18%)
Adherence monitoring of documentation policy for dose, duration, indication	12 (16%)
Providing education to clinicians and other relevant staff on improving antimicrobial use	8 (11%)
